# Supplementary material for: Clearance of maternal barriers by paternal miR159 to initiate endosperm nuclear division in Arabidopsis
Source: Nat Commun. 2018 Nov 27;9:5011. doi: 10.1038/s41467-018-07429-x (PMC6258693; doi:10.1038/s41467-018-07429-x)
Supplement: Supplementary file 1 — Supplementary Information [file 41467_2018_7429_MOESM1_ESM.pdf]

Supplementary Figures and Figure legends

Supplementary Fig. 1 Reproductive phenotypic analysis of Col-0 and the *mir159abc* mutant.

(a) Images of dry seeds from ♀Col-0 ×♂Col-0, ♀Col-0 ×♂*mir159abc*, ♀*mir159abc* ×♂Col-0, ♀*mir159abc* ×♂*mir159abc*, respectively. Statistical analysis of seed size for each category was shown in **Fig. 1c**.

(b) Mature pollen of Col-0 and the *mir159abc* triple mutant by DAPI staining. >500 pollen was observed, and representative images were shown. The yellow arrows indicate twin sperm nuclei. Pollen is dark blue due to auto-fluorescence. Scale bars, 200 μm.

(c) *In vivo* pollen tube growth, guidance and burst in Col-0 and the *mir159abc* triple mutant. Pollen tubes were examined by ovule clearing and decolorized aniline blue staining. >10 pollinated pistils for each genotype were observed, and representative images were shown. Arrowheads indicate the entrance location of pollen tubes in the ovule. Scale bars, 200 μm.

(d) Whole-mount microscopy of the mature ovule of *mir159abc*. Ovules were dissected from fixed and cleared ovaries, and imaged by DIC analysis. >100 ovules were analyzed. Synergid cells, the egg cell, and the central cell are shown by arrows, and the yellow dashed circle surrounds three antipodal cells. The outline of the embryo sac was indicated by the white dashed line. Scale Bar, 20 μm.

Zhao_Fig S1

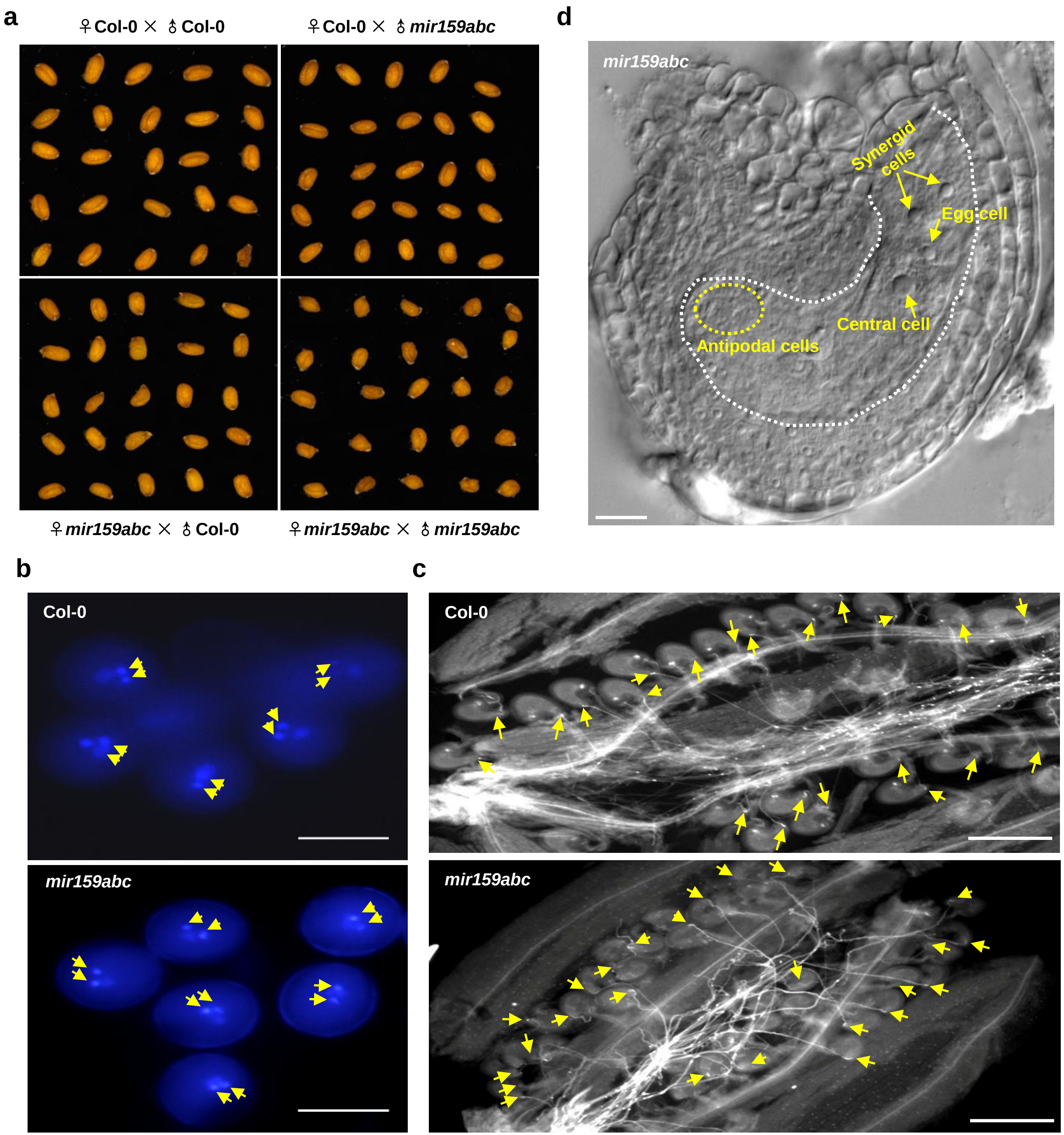
a
♀Col-0 × ♂Col-0
♀Col-0 × ♂mir159abc
♀mir159abc × ♂Col-0
♀mir159abc × ♂mir159abc
d
mir159abc
Synergid cells
Egg cell
Central cell
Antipodal cells
b
Col-0
mir159abc
c
Col-0
mir159abc

Supplementary Fig. 2 Endosperm and embryo development in the loss of paternal miR159.

(a-c) Representative images showing delayed endosperm nuclear divisions at 12 HAP **(a)**, 24 HAP **(b)**, and 48 HAP **(c)** in the loss of paternal miR159. White stars indicate endosperm nuclei or the undivided central cell nucleus. Yellow stars indicate the fused nucleus by the egg cell and the sperm.

(d) About 3-4 week-old seedling morphology and silique length of Col-0, and all *mir159* single, double, and triple mutants. Scale bar, 1 cm.

(e) Representative images showing the progression of endosperm nuclear divisions at 24 HAP when the *mir159ab* double mutant was used as the male. White stars indicate endosperm nuclei or the undivided central cell nucleus. Yellow stars indicate the fused nucleus by the egg cell and the sperm.

(f, g) Embryo development of F1 progenies from either Col-0 **(f)** or *mir159abc* **(g)** as the male. Developing seeds at different DAP (Day After Pollination) from indicated crosses by hand-pollination were observed by DIC imaging. >50 seeds were examined for each genotype. n indicates total numbers of analyzed developing seeds. % indicates the ratio of numbers of developing seeds similar to the representative image relative to total analyzed developing seeds. Scale bar, 20 μm. Source data are provided as a Source Data file.

Zhao_Fig S2

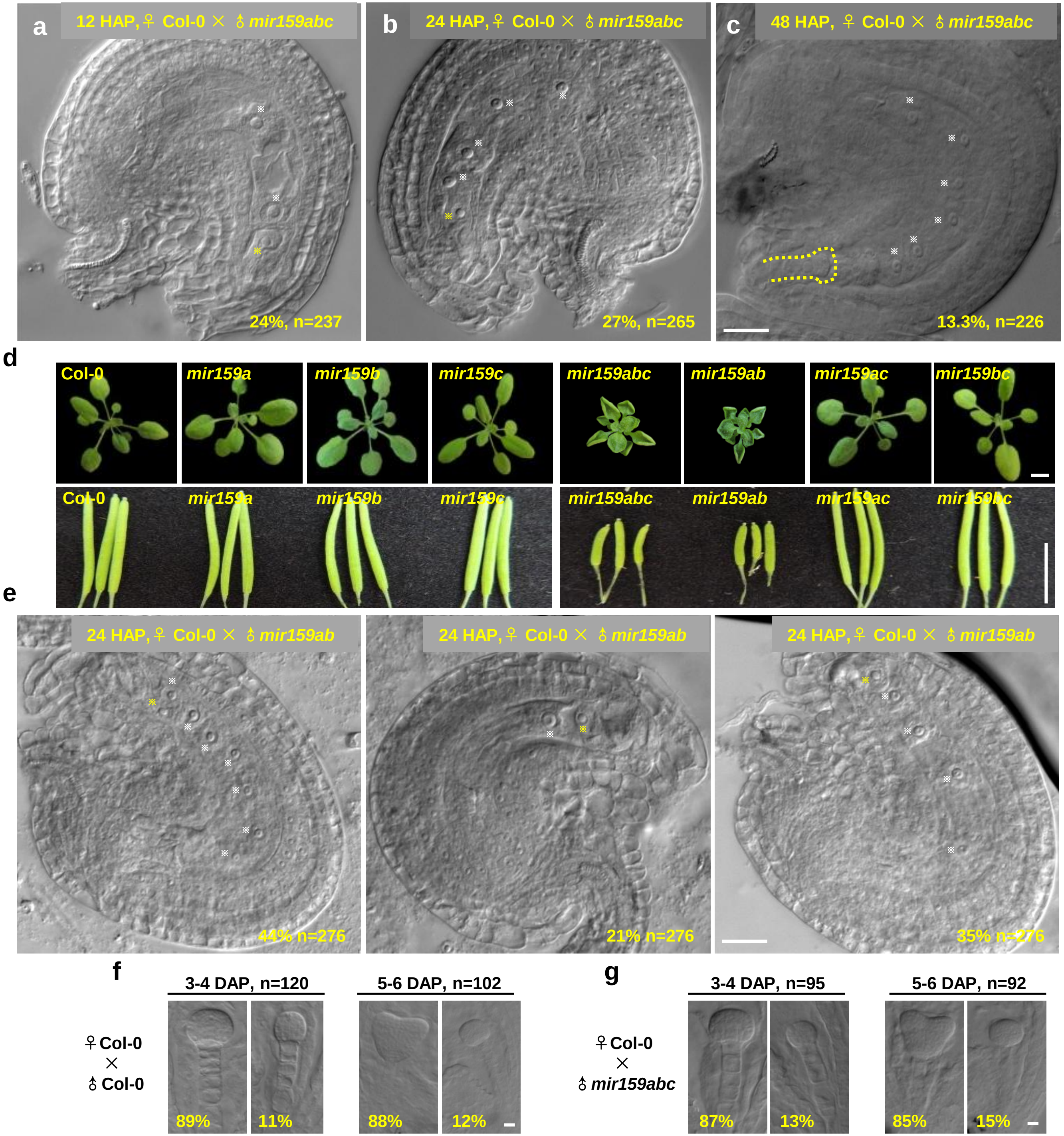
a
12 HAP, ♀ Col-0 × ♂ mir159abc
24%, n=237
b
24 HAP, ♀ Col-0 × ♂ mir159abc
27%, n=265
c
48 HAP, ♀ Col-0 × ♂ mir159abc
13.3%, n=226
d
Col-0
mir159a
mir159b
mir159c
mir159abc
mir159ab
mir159ac
mir159bc
Col-0
mir159a
mir159b
mir159c
mir159abc
mir159ab
mir159ac
mir159bc
e
24 HAP, ♀ Col-0 × ♂ mir159ab
44% n=276
24 HAP, ♀ Col-0 × ♂ mir159ab
21% n=276
24 HAP, ♀ Col-0 × ♂ mir159ab
35% n=276
f
3-4 DAP, n=120
5-6 DAP, n=102
♀ Col-0
×
♂ Col-0
89%
11%
88%
12%
g
3-4 DAP, n=95
5-6 DAP, n=92
♀ Col-0
×
♂ mir159abc
87%
13%
85%
15%

Supplementary Fig. 3 Expression of miR159 targets and subcellular localization of MYB65 in mature pollen and the developing seeds.

(a, b) qRT-PCR analysis of miR159 targets in mature pollen **(a)** and unfertilized mature ovules **(b)** of Col-0 and *mir159abc*, respectively. Biological replicates for each gene are shown by dots. Error bars show SD calculated from three biological replicates.

(c) Subcellular localization of proMYB33::MYB33-GFP and proMYB65::MYB65-RFP in mature pollen. The auto-fluorescence images of pollen for each transgenic plants were shown in the middle panels, and the overlay was performed. >200 pollen were examined for each.

(d) Subcellular localization of proMYB65::MYB65-RFP in the developing seeds. Upper panels indicate fluorescence images of the developing seeds from proMYB65::MYB65-RFP transgenic plants. Lower panels show MYB65-RFP signal quantification corresponding to the image from the upper panel. Arrows were used for RFP signal intensity quantification, and the measurements were performed using the Image-Pro Insight software. Numbers shown in the Y-axis indicate signal intensity along the arrow. Dashed circles indicate MYB65-RFP signals. Embryo sacs are red due to auto-fluorescence. n represents total numbers of analyzed developing seeds for each category, and % indicates the ratio of developing seeds similar to the representative image relative to total ones. Source data are provided as a Source Data file.

Zhao_Fig S3

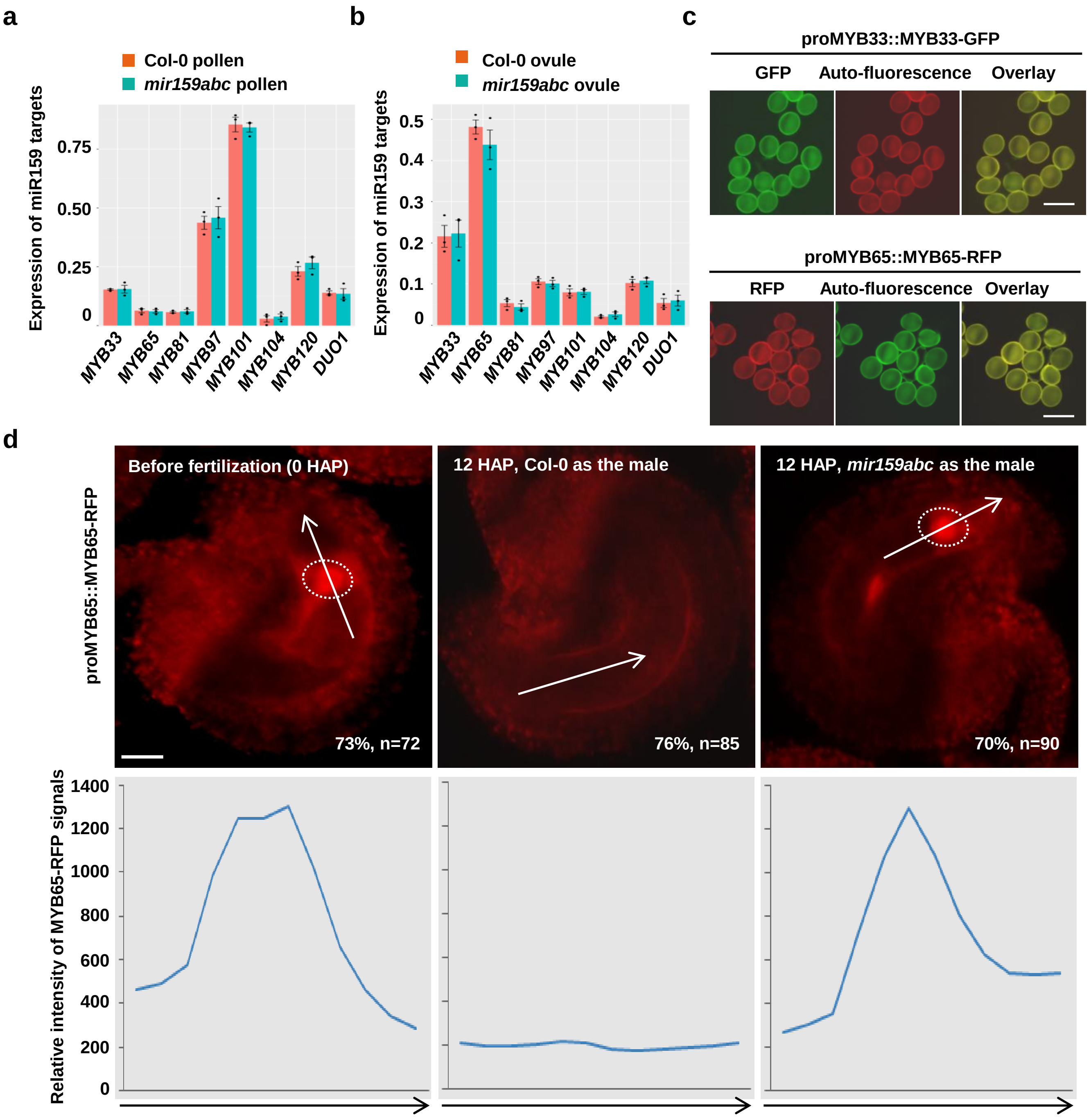
a
Col-0 pollen
mir159abc pollen
Expression of miR159 targets
0.75
0.50
0.25
0
MYB33
MYB65
MYB81
MYB97
MYB101
MYB104
MYB120
DUO1
b
Col-0 ovule
mir159abc ovule
Expression of miR159 targets
0.5
0.4
0.3
0.2
0.1
0
MYB33
MYB65
MYB81
MYB97
MYB101
MYB104
MYB120
DUO1
c
proMYB33::MYB33-GFP
GFP
Auto-fluorescence
Overlay
proMYB65::MYB65-RFP
RFP
Auto-fluorescence
Overlay
d
proMYB65::MYB65-RFP
Before fertilization (0 HAP)
73%, n=72
12 HAP, Col-0 as the male
76%, n=85
12 HAP, mir159abc as the male
70%, n=90
Relative intensity of MYB65-RFP signals
1400
1200
1000
800
600
400
200
0

Supplementary Fig. 4 Expression of three *MIR159* genes in the developing seeds.

(a-c) Promoter activities of *MIR159a* **(a)**, *MIR159b* **(b)**, and *MIR159c* **(c)** in the developing seeds. Fluorescence images of the proMIR159::NLS-GFP/proFWA::RFP doubly transgenic plants in the unfertilized ovule (top panels), the developing seed at 12 HAP (middle panels), and the developing seed after 24 HAP (bottom panels). White dashed circles indicate the RFP signal, and the white arrowheads indicate the GFP signal. proFWA::RFP was used as the central cell/endosperm marker. n represents total numbers of analyzed developing seeds for each genotype, and % indicates the ratio of developing seeds similar to the representative image relative to total ones. Scale bar, 20 µm.

(d) GFP signal quantification. The measurements were described as **Fig. 3d**. One dot indicates the result from one image. Error bars show SD calculated from 10 developing seeds for each category. Source data are provided as a Source Data file.

Zhao_Fig S4

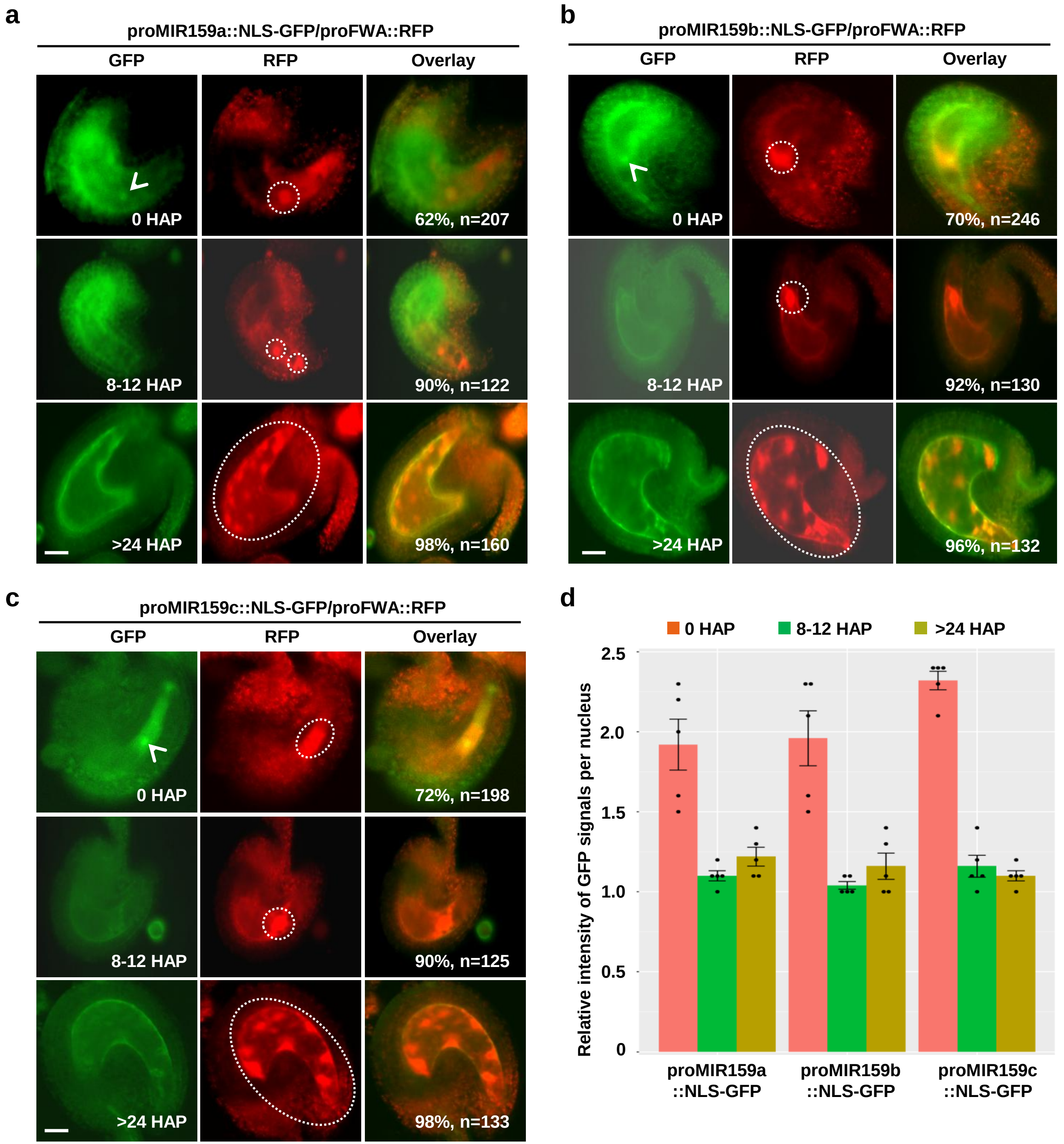
a
proMIR159a::NLS-GFP/proFWA::RFP
GFP
RFP
Overlay
0 HAP
62%, n=207
8-12 HAP
90%, n=122
>24 HAP
98%, n=160
b
proMIR159b::NLS-GFP/proFWA::RFP
GFP
RFP
Overlay
0 HAP
70%, n=246
8-12 HAP
92%, n=130
>24 HAP
96%, n=132
c
proMIR159c::NLS-GFP/proFWA::RFP
GFP
RFP
Overlay
0 HAP
72%, n=198
8-12 HAP
90%, n=125
>24 HAP
98%, n=133
d
0 HAP
8-12 HAP
>24 HAP
Relative intensity of GFP signals per nucleus
2.5
2.0
1.5
1.0
0.5
0
proMIR159a ::NLS-GFP
proMIR159b ::NLS-GFP
proMIR159c ::NLS-GFP

Supplementary Fig. 5 Over-expression of *mMYB33* caused reduced seed set.

(a) proFWA::MYB33-YFP (MYB33-OE) and proFWA::mMYB33-YFP (mMYB33-OE) in the developing seeds pollinated by Col-0 or *mir159abc*. White arrows indicate GFP signals. Auto-fluorescence of embryo sacs from the wild type plants are shown on the left for each genotype. n represents total numbers of observed developing seeds for each genotype, and % indicates the ratio of developing seeds similar to the representative image relative to total ones. Scale bar, 20 μm.

(b, c) Quantification of GFP signals in **(a)**. The measurements were described as **Fig. 3d**. One dot indicates the result from one image. Error bars show SD calculated from 10 developing seeds for each category.

(d) Statistical analysis of reduced seed set in mMYB33-OE transgenic plants. Numbers in the X-axis indicate individual transgenic plants of mMYB33-OE. One dot indicates the result from one silique. Error bars show SD calculated from ~10 mature seeds for each individual transgenic line. Source data are provided as a Source Data file.

Zhao_Fig S5

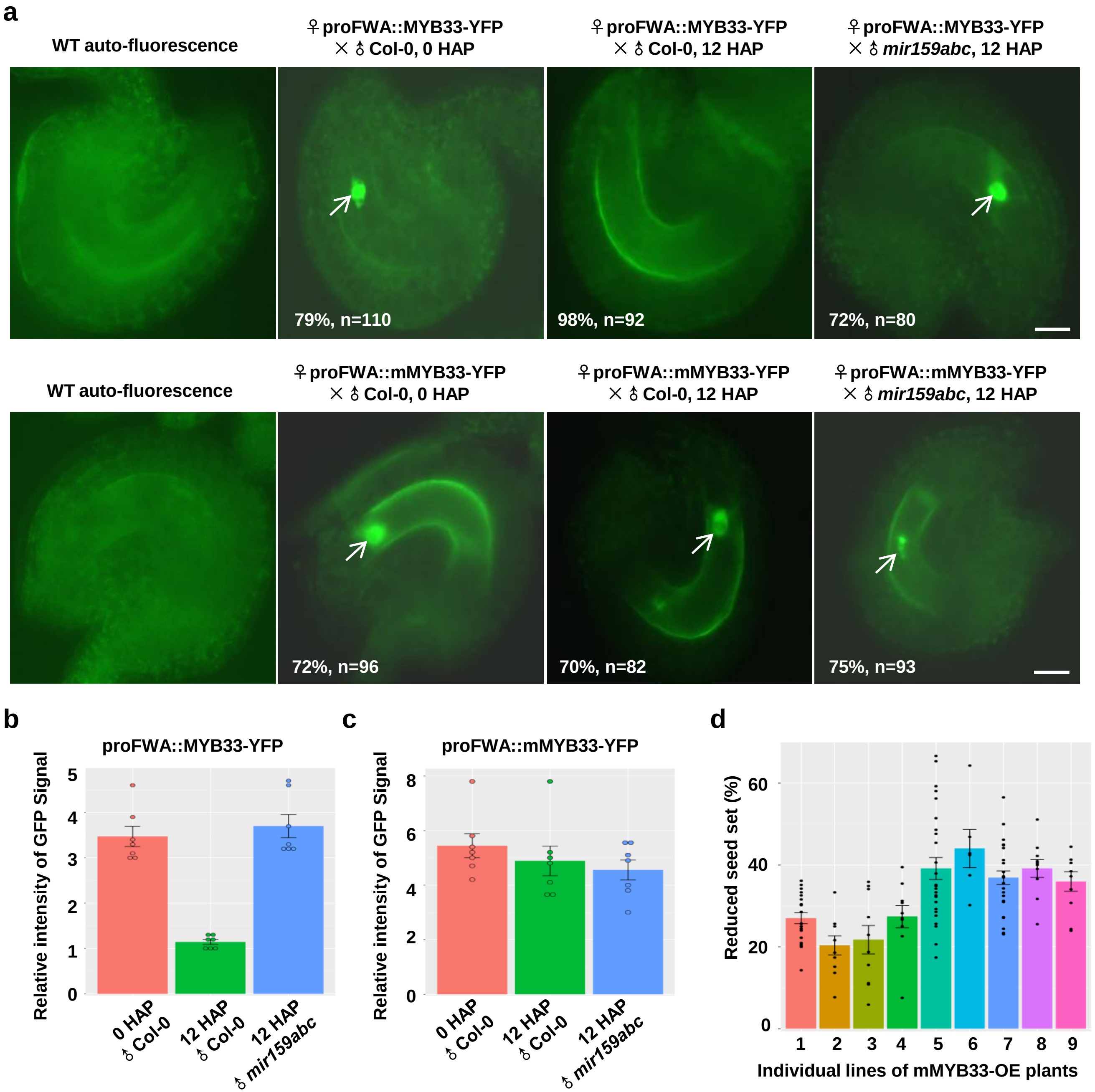
a
WT auto-fluorescence
♀proFWA::MYB33-YFP × ♂Col-0, 0 HAP
♀proFWA::MYB33-YFP × ♂Col-0, 12 HAP
♀proFWA::MYB33-YFP × ♂*mir159abc*, 12 HAP
79%, n=110
98%, n=92
72%, n=80
WT auto-fluorescence
♀proFWA::mMYB33-YFP × ♂Col-0 HAP
♀proFWA::mMYB33-YFP × ♂Col-0, 12 HAP
♀proFWA::mMYB33-YFP × ♂*mir159abc*, 12 HAP
72%, n=96
70%, n=82
75%, n=93
b
proFWA::MYB33-YFP
Relative intensity of GFP Signal
0
1
2
3
4
5
0 HAP ♂Col-0
12 HAP ♂Col-0
12 HAP ♂*mir159abc*
c
proFWA::mMYB33-YFP
Relative intensity of GFP Signal
0
2
4
6
8
0 HAP ♂Col-0
12 HAP ♂Col-0
12 HAP ♂*mir159abc*
d
Reduced seed set (%)
0
20
40
60
1 2 3 4 5 6 7 8 9
Individual lines of mMYB33-OE plants

Supplementary Tables

Supplementary Table 1. Reduced seed set in the *mir159abc* triple mutant.

Female × Male	♀Col-0 × ♂Col-0	♀Col-0 × ♂*mir159abc*	♀*mir159abc* × ♂Col-0	♀*mir159abc* × ♂*mir159abc*
Normal seeds	7380	4721	3894	3184
aborted seeds	150	2542	1917	2502
Seed set	98%	65%	67%	56%

The average number of seeds per Col-0 silique was 40~50, while the average number of the *mir159abc* mutant was 30-40. ~150 siliques from >50 plants were examined for each genotype.

Supplementary Table 2. Transmission efficiency (TE) of the *mir159abc* triple mutant.

Parental genotype (female × male)		Genotype of F1 plants		
		-/+	WT	TE
Col-0 × *mir159abc*/+	*mir159a*/+	78	148	34.5%
	mir159b/+	66	160	29.2%
	mir159c/+	72	154	31.9%
mir159abc/+ × Col-0	*mir159a*/+	48	105	31.4%
	mir159b/+	31	122	20.3%
	mir159c/+	41	112	26.8%

Genotyping was examined by PCR-based methods using specific primers corresponding to the T-DNA insertion location in *MIR159a*, *b*, *c*, respectively. ~10 pistils were hand-pollinated.

Supplementary Table 3. Primer sequences used in this study.

**Oligo Name**	**5'-3' sequence**	**Use**
MYB33F1	gccctcgagtttacaagttattttg	amplify

		genomic DNA
MYB33R1	ggcactagtgggtagttctgtcatttg	amplify genomic DNA
MYB65F1	cgcggtacccgttgaaagatatatgtt	amplify genomic DNA
MYB65R1	gccgtcgaccagcgaccaaacaggag	amplify genomic DNA
MYB33F2	cgcctcgagatgagttacacgagcac	amplify CDS
MYB33R2	ggcgtcgacgggtagttctgtcatttg	amplify CDS
MYB33F3	ggggcagtgaagctggaactgccaagcttgcaatattcagaaac	mMYB33
MYB33R3	gtttctgaatattgcaagcttggcagttccagcttcactgcccc	mMYB33
FWAF1	CAAAACCCAGAAACTGAAATCGACTTG	Amplify promoter
FWAR1	TTTCCCTCAATGCAATAACCTGGACA	Amplify promoter
MYB33F4	TGTATCCCAAACTAAATCCGAGG	qRT-PCR, 5' RACE
MYB33R4	CGTCAGGCCTTGTTATATCCAAG	qRT-PCR, 5' RACE
MYB65F2	AGATTACAAGTCCCTCGCAAC	qRT-PCR, 5' RACE
MYB65R2	CAAGTAAAACATCAGGCCGTG	qRT-PCR, 5' RACE
MYB81F1	TCGCCTTCGTTGGGTAAATC	qRT-PCR
MYB81R1	CATTATCTGTTCGTCCCGGTAG	qRT-PCR
MYB97F1	ATCATACAACTCCACTCTCAGC	qRT-PCR
MYB97R1	CTCGTTATCTGTTCTGCCTGG	qRT-PCR
MYB101F1	GGCGGACTCTTCAAGGAC	qRT-PCR
MYB101R1	GTTGTGAATATTAGGGTTTGCTCC	qRT-PCR

MYB104F1	CGATGAGGAAGAGAAGCGTG	qRT-PCR
MYB104R1	ACCCTTTCCTTTCAATCGCA	qRT-PCR
MYB120F1	GTAACAAATGGGCTCGCATG	qRT-PCR
MYB120R1	GGATGGAGTTGATGGTTAGGG	qRT-PCR
DUO1F1	CTGACGAAGAGAGGACTGTG	qRT-PCR
DUO1R1	AGATTTGGGATTGAAACTCG	qRT-PCR
N-UBQ5	GGTGCTAAGAAGAGGAAGAAT	qRT-PCR
C-UBQ5	CTCCTTCTTTCTGGTAAACGT	qRT-PCR
GeneRacer RNA Oligo	cgacuggagcacgaggacacugacauggacugaaggaguagaaa	5’RACE
GeneRacer™ 5’Primer	CGACTGGAGCACGAGGACACTGA	5’RACE
GeneRacer™ 5’NestedPrimer	GGACACTGACATGGACTGAAGGAGTA	5’RACE
MYB65Race	CTTGACCTGATGATGACCCAACAGTCACGTAACTG	5’RACE
MYB65F3	GACGATCTAAATTTTGCTGCTAG	5’RACE
MYB33Race	GACAGACAGGTGGCATGTTGCTCCAAGAACAAGAC	5’RACE
MYB33F5	GTTGTGAATCTAATGTCTTC	5’RACE
MYB33F6	AGTTGTTGTATCCTGGGTGTAGCA	5’RACE
GFPRace	AGCTCCTCACCCTTGCTCACCATTGATATCACCAC	5’RACE
GFPR1	TAGTTGCCGTCGTCCTTGAA	5’RACE
