# Supplementary material for: Clearance of maternal barriers by paternal miR159 to initiate endosperm nuclear division in Arabidopsis
Source: Nat Commun. 2018 Nov 27;9:5011. doi: 10.1038/s41467-018-07429-x (PMC6258693; doi:10.1038/s41467-018-07429-x)

Source Data 1. Original images of F1 siliques from 6 DAP (Day After Pollination) ♀Col-0 × ♂Col-0 (the top panel) and ♀Col-0 × ♂*mir159abc* (three lower panels), respectively. Pictures were taken under same conditions.

Zhao et al_Source data 1

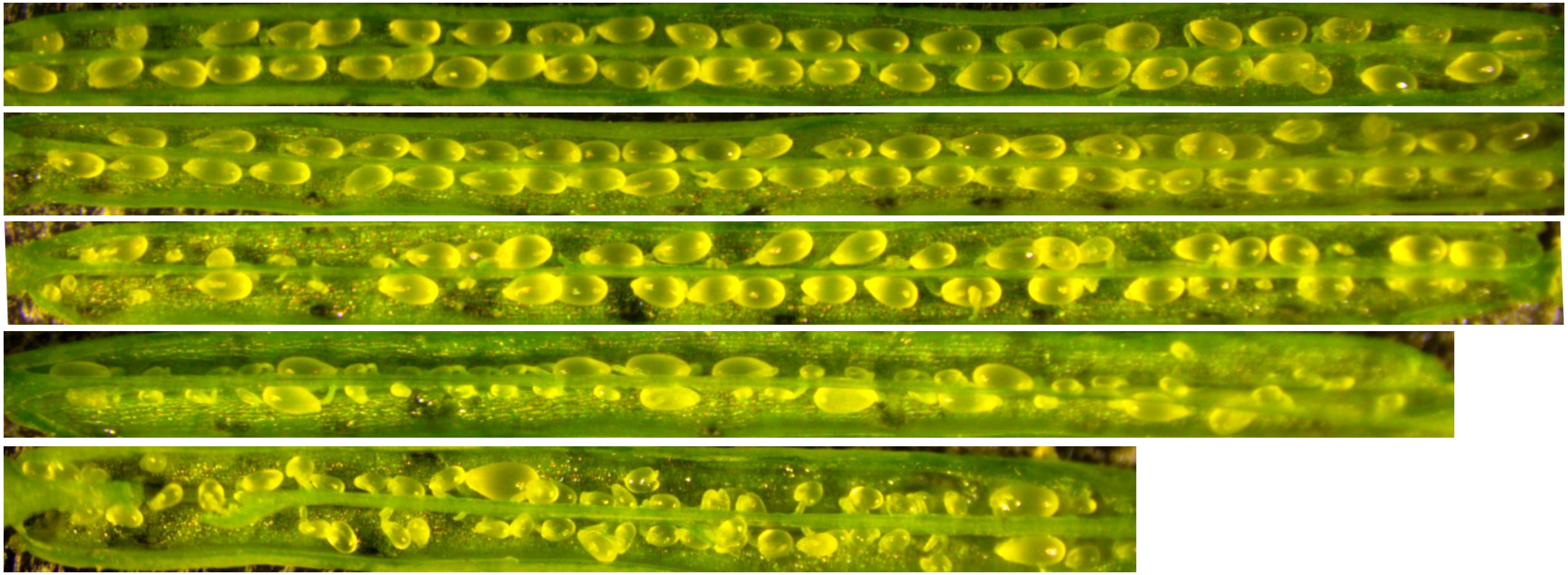

Source Data 2. Original images of detection of 3'cleavage products of *MYB33* and *MYB65* by 5'RACE-PCR. PCR products from 5'RACE experiments were analyzed on agarose gels, and original uncropped gel images are shown. Other unlabelled lanes are samples unrelated with this study.

Zhao et al_Source data 2

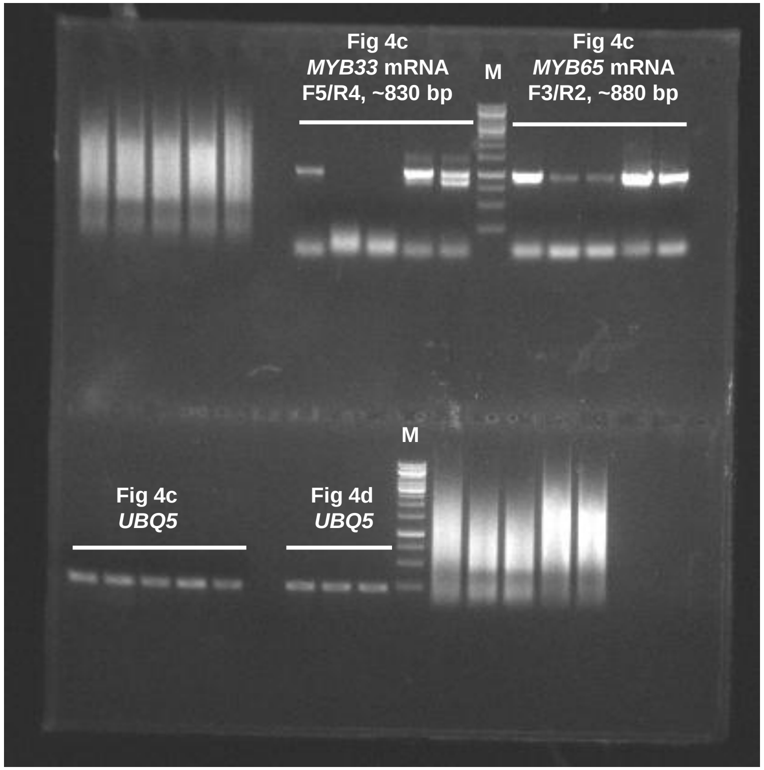

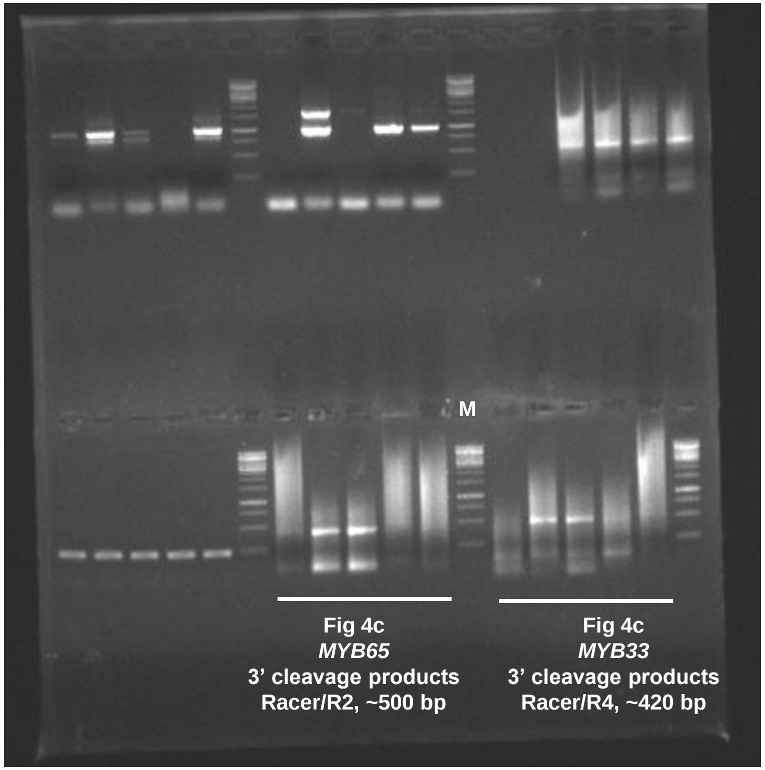

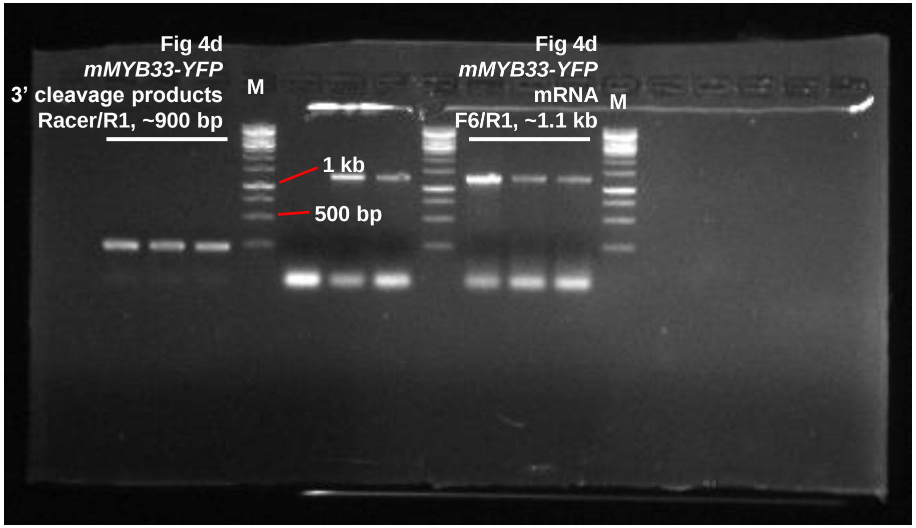

Source Data 3. Original images of dissected seeds from MYB33-OE or mMYB33-OE transgenic plants.

Zhao et al_Source data 3

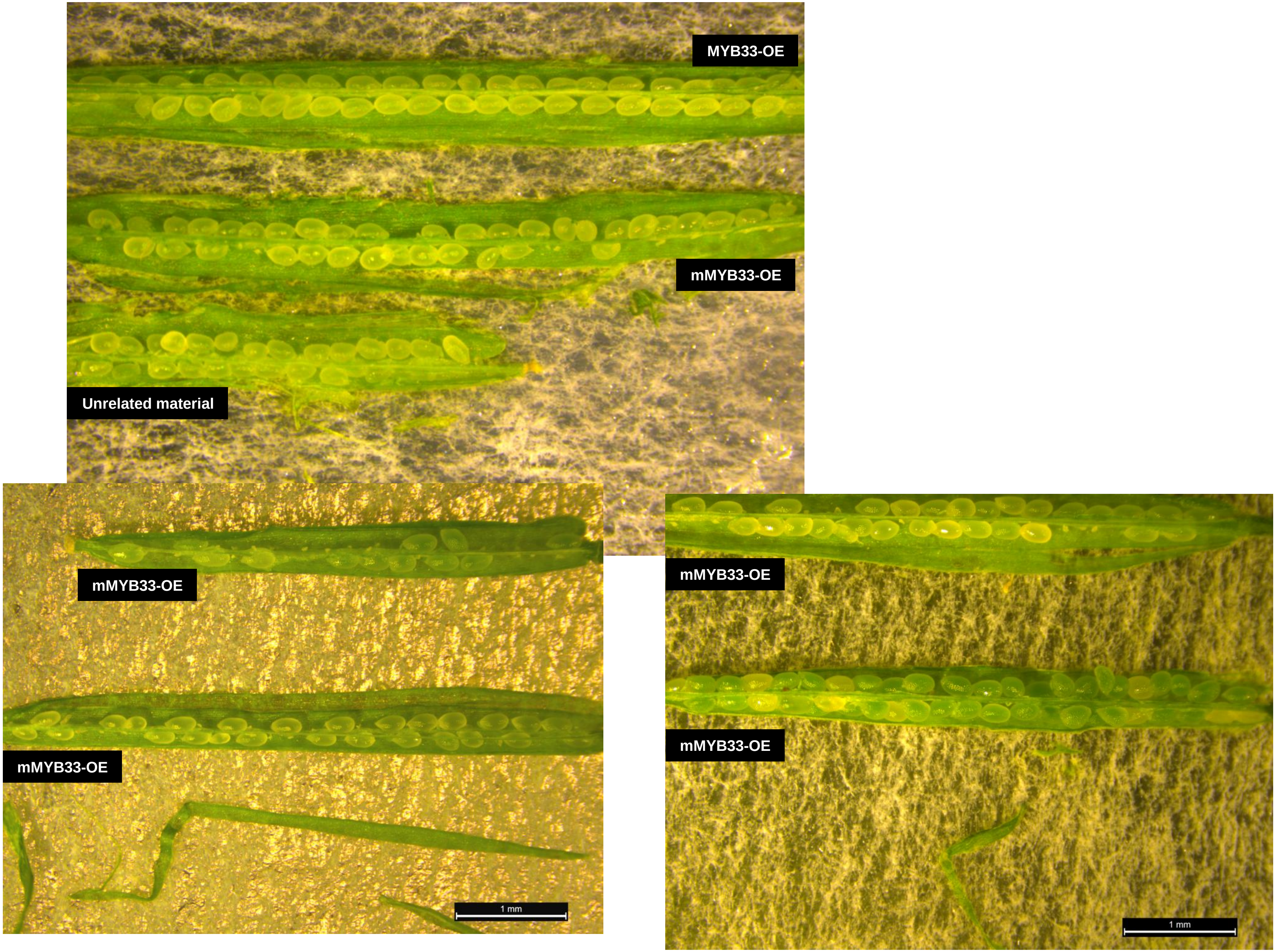

Supplement: Supplementary file 3 — Source Data [file 41467_2018_7429_MOESM3_ESM.zip › siliques_RACR_PCR.pdf]
